# Chemical Composition and Biological Activity of Essential Oil from Leaves and Fruits of Limoncillo (*Siparuna muricata* (Ruiz & Pav.) A. DC.)

**DOI:** 10.3390/antibiotics12010082

**Published:** 2023-01-03

**Authors:** Vladimir Morocho, Mariangel Hidalgo-Tapia, Israel Delgado-Loyola, Luis Cartuche, Nixon Cumbicus, Eduardo Valarezo

**Affiliations:** 1 Departamento de Química, Universidad Técnica Particular de Loja, Loja 110150, Ecuador; 2 Carrera de Bioquímica y Farmacia, Universidad Técnica Particular de Loja, Loja 110150, Ecuador; 3 Departamento de Ciencias Biológicas y Agropecuarias, Universidad Técnica Particular de Loja, Loja 110150, Ecuador

**Keywords:** *Siparuna muricata*, essential oil, chemical composition, antimicrobial activity, antioxidant activity, anticholinesterase activity

## Abstract

*Siparuna muricata* is an aromatic native shrub or tree from Ecuador known as “limoncillo” or “limón de la sierra” due to its citrus odor. In this study, the chemical composition and biological activity of essential oil from the leaves and fruits of this species was determined. The essential oil was isolated by subjected to hydrodistillation. The chemical composition was determined by gas chromatography equipped with a flame ionization detector and gas chromatography coupled to mass spectrometry. The enantiomeric distribution was determined by gas chromatography using an enantioselective column. The antimicrobial activity against three Gram-positive bacteria, two Gram-negative bacteria and two fungi was determined by the broth microdilution method. The antioxidant activity was analyzed using the 2,2-diphenyl-1-picrylhydryl free radical and 2,2’-azinobis-3-ethylbenzothiazoline-6-sulfonic acid radical cation scavenging activity of essential oil. The acetylcholinesterase inhibitory effect of the essential oil was measured using a spectrophotometric method. The yield was 1.2 ± 0.1 mL/kg for leaves and 1.8 ± 0.2 mL/kg for fruits. A total of 51 compounds were identified in the leaves of the essential oil and 41 in the fruits. In both cases, the chemical compositions were dominated by the group of monoterpene hydrocarbons compounds. The main compound was α-pinene with 23.22 ± 1.03% in essential oil of the leaves and limonene with 24.92 ± 1.20% in the fruits. In both essential oils, five pairs of enantiomers with different enantiomeric excesses were identified. The essential oil of limoncillo leaves presented a strong activity against the fungus *Aspergillus niger* (ATTC 10231) and Gram-positive bacterium *Enterococcus faecium* (ATCC 27270) with a MIC of 250 μg/mL and 500 μg/mL, respectively. The essential oil from fruits and leaves of *Siparuna muricata* presented a moderate antioxidant activity with the ABTS method with a SC_50_ of 775.3 ± 1.3 µg/mL and 963.3 ± 1.6 µg/mL, respectively. Additionally, the leaves essential oil reported an IC_50_ value of 52.98 ± 1.04 µg/mL and the fruits essential oil an IC_50_ value of 98.84 ± 1.04 µg/mL, which can be considered a very strong anticholinesterase activity.

## 1. Introduction

The aromatic plants or aromatic plant species are known as all kinds of species whose relevance lie upon having some type of aroma [1]. This odor is given by volatile components, which are volatile secondary metabolites which together are called essential oil (EO) or essences. Aromatic plants make up a wide and diverse list of species that have various uses, especially in food and traditional medicine, and as raw materials to extract essential oils [2]. Homemade and in the food industry, aromatic plants are used to make drinks, as condiments or as odorants with the name of fine herbs. The volatile aromatic compounds in the aromatic species have given unique properties to these species, many of which are considered medicinal. Hence, aromatic plants are used in the field of traditional medicine, specially in developing countries [3]. Until the discovery of modern medicine, humanity depended on aromatic and medicinal plants for treating illness [4]. The use of aromatic species is incorporated in customs and traditions of many countries, to the point it still has not been replaced by modern or conventional medicine. According to the World Health Organization, approximately 80% of the population of developing countries still depend on plant produced medicines for their healthcare [5]. Aromatic plants can be found in most genera of various botanical families, such as Apiaceae, Asteraceae, Burseraceae, Cupressaceae, Fabaceae, Lamiaceae, Lauraceae, Myrtaceae, Pinaceae, Piperaceae, Poaceae, Rutaceae, Siparunaceae, Zingiberaceae and so on [6].

Essential oils (EOs), also known as volatile oils, can be found in aromatic plants in which characteristic aromas are concentrated. These oils are located in the vascular system, leaves, stems, flowers, or in other places depending on the plant species; in general, they constitute 0.1 to 3% of the dry weight of the plant, although there are reports of yields close to 6% [7]. Generally, essential oils are liquids, poorly soluble in water, but volatilizable with steam; they evaporate at different rates under atmospheric pressure. Essences are innocuous, if the dose administered does not exceed the toxicity limits [8]. Volatile oils undergo chemical degradation in the presence of sunlight, hot air, strong acids and alkalis, generating oligomers of indeterminate nature. Essential oils can be extracted from plant samples by different methods, such as steam distillation, extraction with volatile solvents and supercritical fluids; the most common being extraction with steam dragging [9]. Plants can produce essential oil for different purposes; on one hand, they protect the plant from pests and diseases and to attract pollinating insects and birds. However, essential oils have diverse and interesting biological properties, such as antifungal, antibacterial, antioxidant, antienzymatic, antiviral, anticancer, antiphlogistic and insect repellent activities [10].

Siparunaceae (A. DC.) Schodde is a small family of angiosperm plants in the order Laurales. A total of 59 species of woody plants belong to this botanical family, most of which contain essential oils. The Siparunaceae family has great economic, medicinal and phytochemical importance. Fruits and leaves of certain species from Siparunaceae are used in traditional medicine to treat gastrointestinal diseases, cough, fever and rheumatism. In addition, this species is used as timber [11]. The species of the Siparunaceae family are distributed in two genera. The genus Glossocalyx Benth. with one species and the genus *Siparuna* with 58 species [12]. The species of the genus *Siparuna* are distributed in South America and Central America. In Ecuador, approximately 40 species of this genus are represented; 11 species have been recorded in the Andean forests [13]. *Siparuna muricata* (Ruiz & Pav.) A. DC. is an aromatic native shrub or tree from Ecuador known as “limoncillo” or “limón de la sierra” due to its citrus odor. This species is widely distributed in Andean Ecuadorian regions between 1000 and 3500 m a.s.l., in this country it can be found especially in the Andean provinces of Azuay, Bolívar, Cañar, Chimborazo, Loja and Tungurahua [14]. In the methanolic extract of this species, alkaloids, coumarins, steroids, flavonoids, cardiac glycosides, sesquiterpene lactone and tannins were found as the main secondary metabolites [15]. In Ecuador, the leaves of *Siparuna muricata* in infusion are used to treat colds and are applied to the armpits to neutralize bad odors [14].

The essential oil of some species of the genus *Siparuna*, such as *Siparuna cymose* [16], *Siparuna echinata*, *Siparuna eggersii* [17], *Siparuna grandiflora* [18], *Siparuna guianensis* [19], *Siparuna schimpffii* [20] and *Siparuna thecaphora* [21], were studied to determine the chemical composition and biological activities. In Ecuador, the essential oil of four species of the *Siparuna* genus has been studied, namely, *Siparuna echinata*, *Siparuna eggersii* [17], *Siparuna schimpffii* [20] and *Siparuna muricata* [11]. In 2021, Burneo et al. studied the chemical composition of the essential oil of *Siparuna muricata* [11] collected in the province of Loja, southern Ecuador. However, to date, the extraction and study of the essential oil from the fruits of this species has not conducted, nor has the biological activity of the oil from the leaves been determined. The fact that the chemical composition and biological activity of the essential oil extracted from the leaves and fruits of *Siparuna muricata* has not been studied, that it is a species widely distributed in the Andean region of Ecuador and it is used in traditional medicine that motivated the authors to conduct this study to isolate and determine the chemical composition and biological activities of the essential oil of the leaves and fruits of *Siparuna muricata*. In this investigation, within the chemical composition, the majority and minority compounds and the enantiomeric distribution of the essential oil isolated from the leaves and fruits of the species *Siparuna muricata* are determined, and in the biological activity tests the antibacterial, antifungal, antioxidant and anticholinesterase activities are analyzed.

## 2. Results

### 2.1. Essential Oil Isolation

The essential oil of *Siparuna muricata* isolated by hydrodistillation presented a characteristic citrus odor and greenish color. The yield was 0.12 ± 0.01% (*v*/*w*) or 1.2 mL/kg for leaves and 0.18 ± 0.02% (*v*/*w*, 1.8 mL/kg) for fruits.

### 2.2. Chemical Composition of Essential Oil

**Figure 1:**
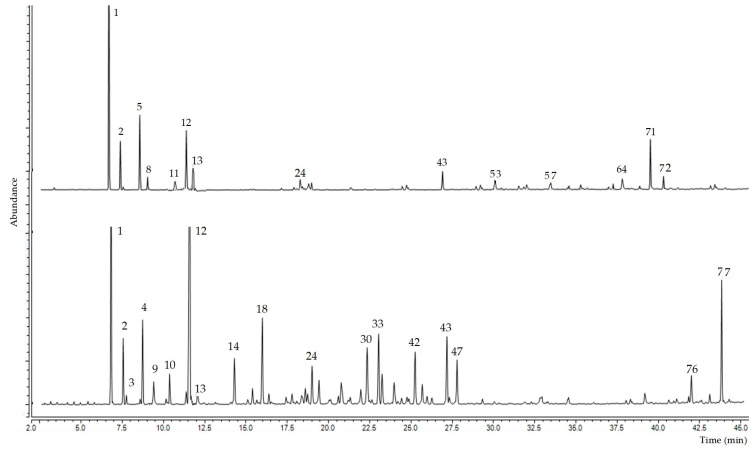
Representative chromatograms of the essential oil from leaves (upper) and fruits (lower) of *Siparuna muricata*. The numbers correspond to the compound numbers in Table 1.

Using gas chromatography coupled with mass spectrometry, it was possible to identify 77 chemical compounds in the essential oil samples from the leaves and fruits of *Siparuna muricata*, representative chromatograms of the 2 oils are shown in Figure 1.

In the leaves of the essential oil, 51 compounds were identified, which represent 97.16% of the total. In the fruit essential oil, 41 compounds were identified, which represent 90.13% of the percentage of the chemical composition (Table 1). In both cases, the chemical compositions were dominated by the group of hydrocarbon monoterpene compounds, although with different percentages, 51.16% for the leaves and 45.84% for the fruits. The second most representative group of compounds was the oxygenated sesquiterpenes (22.97%) in the leaves and the oxygenated monoterpenes (25.07%) in the fruits. Compounds belonging to the group of diterpenes hydrocarbons were not detected in the leaves.

**Figure 2:**
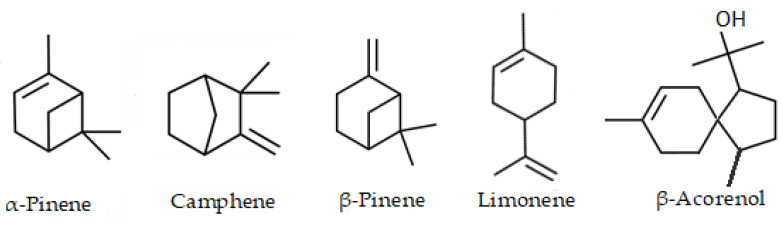
Chemical structures of main compounds of the essential oil from leaves in fruits of *Siparuna muricata*.

The major compound was pinene <α-> (mixture of (+) and (−) enantiomers) with 23.22 ± 1.03% in the essential oil of the leaves and limonene (mixture of (+) and (−) enantiomers) with 24.92 ± 1.20% in the fruits. Additionally, acorenol <β-> (12.71%), pinene <β-> (9.47%), limonene (8.71%) and camphene (5.17%) was found as the main (>5%) compounds in the leaves essential oil. In the fruits, in addition to limonene, pinene <α-> (10.90%) was determined as the main compound. The chemical structures of the main compounds are shown in Figure 2.

### 2.3. Enantiomeric Analysis

The enantiomeric composition of leaves and fruits from *Siparuna muricata* essential oil was achieved for the first time. Table 2 shows the enantiomers, retention indices, enantiomeric distribution, and enantiomeric excess (e.e., in each pair of compounds) of both essential oils.

**Figure 3:**
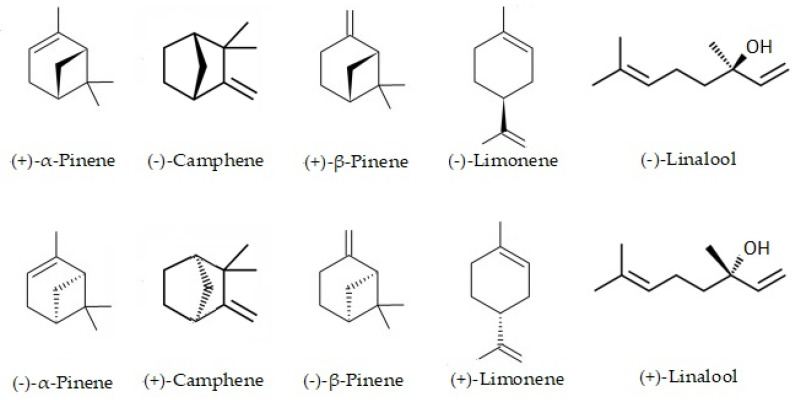
Chemical structures of the stereoisomers of the essential oil from leaves in fruits of *Siparuna muricata*.

Using a chiral column could be quantified by five pairs of enantiomers, whose peaks were well separated at the base. The chemical structures of the stereoisomers are shown in Figure 3. The (−)-camphene was found pure in essential oil from leaves, with 100% enantiomeric excess, while in fruits, essential oil (−)-α-pinene was shown to be the only enantiomer (e.e. = 100%). (+)-α-pinene and (−)-α-pinene in leaves essential oil and (+)-limonene and (−)-limonene in fruit essential oil were found in a racemic mixture (e.e. near 50%).

### 2.4. Antimicrobial Activity

The antibacterial and antifungal activities of essential oil from the leaves and fruits of limoncillo were determined by the microdilution broth method. Ampicillin, ciprofloxacin and amphotericin B were used as a positive control and dimethylsulfoxide as a negative control. The maximum evaluated concentration was 4000 µg/mL. Table 3 shows the minimum inhibitory concentration (MIC) values of the essential oil against the tested microorganisms. Three Gram-positive, two Gram-negative bacteria, and two fungi are used in the assays. The *Siparuna muricata* leaves essential oil reported MIC values of 500 µg/mL against *Enterococcus faecium* (ATCC 27270) and 250 µg/mL against *Aspergillus niger* (ATTC 10231). The *Siparuna muricata* fruit essential oil reported MIC values of 1000 µg/mL against fungi *Candida albicans* (ATTC 10231) and *Aspergillus niger* (ATTC 10231).

### 2.5. Antioxidant Activity

Two methods ABTS and DPPH were used to determine the antioxidant activity of essential oil from the leaves and fruits of limoncillo, the results obtained are shown in Table 4. The scavenging capacity (SC_50_) was used to report the results. SC_50_ is the concentration of the essential oil that scavenges or decreases the concentration of the radical at 50%. The maximum evaluated concentration was 4000 µg/mL and trolox was used as a positive control. The fruit essential oil presented a SC_50_ of 775 µg/mL with method ABTS, but did not report activity at the maximum concentration tested (4000 µg/mL).

### 2.6. Anticholinesterase Activity

**Figure 4:**
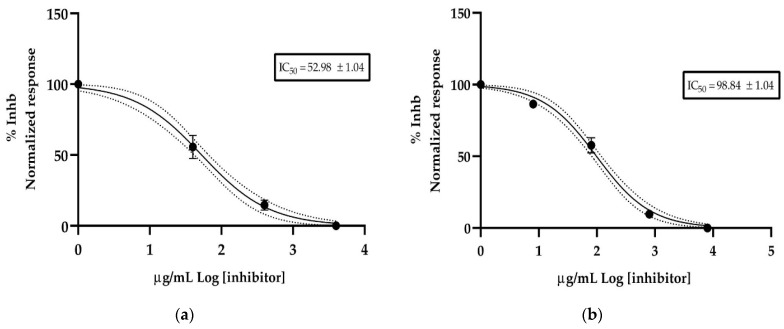
Half-maximum inhibitory concentration of S. *muricata* essential oil against acethylcholinesterase, (**a**) essential oil isolated from leaves and (**b**) essential oil isolated from fruits.

Three concentrations of essential oil from the leaves and fruits of *Siparuna muricata* were used to determine its anticholinesterase activity. Figure 4 shows the rate of reaction of acetylcholinesterase (AChE) against essential oil. The results plotted as Log of concentration of essential oil vs. normalized response rate of reaction allowed us to calculate the half-maximal inhibitory concentration (IC_50_) value. The leaves essential oil reported an IC_50_ value of 52.98 ± 1.04 µg/mL and fruits essential oil an IC_50_ value of 98.84 ± 1.04 µg/mL. The donepezil (positive control) exhibited an IC_50_ value of 12.40 ± 1.35 µg/mL.

## 3. Discussion

The yield of the fruits was higher than of the leaves, even so, according to the categorization proposed by the Agency of Ciencia y Tecnología para el Desarrollo (CYTED), the yields of the leaves and fruits of *Siparuna muricata* are low, given that values less than 5 mL/kg are considered low, values between 5 mL/kg and 10 mL/kg intermediate and values greater than 10 mL/kg high [22]. The yield of essential oil is variable between the parts of the plant because each plant organ has its own morphology and plant organelles where different amounts of essential oil are stored. In this context, the fruits usually present higher yields than the leaves [23]. The yield value is information particularly used as a criterion for selecting potentially marketable aromatic species. However, it must be analyzed together with other characteristics of the essential oil, such as the originality of the aroma, chemical composition and pharmacological effects. In the case of the essential oil of *Siparuna muricata*, it is worth noting its strong, persistent and distinctive aroma of lemon. Numerous examples of species with relatively low yields are recognized and exploited for their cosmetic, seasoning and medicinal qualities, such as *Achillea millefolium* L. [24], *Aloysia citriodora* Palau [25], *Mentha spicata* L. [26], *Tagetes minuta* L., and others.

The essential oil of the leaves and fruits presented different compositions of the seventy-seven compounds, only 15 were found in both oils. Of these 15 only 2 the pinene <α-> and the limonene are main compounds. The main compound in leaves were α-pinene, acorenol <β->, pinene <β->, limonene and camphene, and limonene and pinene <α-> were the main compounds in essential oil fruit, this can be explained because many factors influence the chemical composition of essential oil, these factors can be classified into two groups, namely, intrinsic and extrinsic factors, and within the intrinsic factors is the part of the plant used for isolating essential oil [27]. This is the first report on the chemical composition of the essential oil of *Siparuna muricata* fruits. However, information on essential oil extracted from fruits of other species of the *Siparuna* genus can be found in the literature. Thus, in the essential oil from the fruit of *Siparuna guianensis* collected in Brazil the main constituents were 2-undecanone (32.5%), β-pinene (19.6%) and limonene (13.6%) [28]. The major components in essential oil of fruits from *Siparuna thecaphora* collected in Costa Rica are 2-undecanone (18.7%), geranial (14.4%), neral (10.7%), citronellal (8.4%), citronellol (6.3%) and β-pinene (6.5%) [29].

Regarding the antimicrobial activity, Van Vuuren and Holl proposed that for essential oil an MIC ≤ 100 µg/mL is very strong activity, from 101 to 500 µg/mL the activity is strong, a moderate activity is when value lies between 501 to 1000 µg/mL and activities > 1001 µg/mL are considered inactive [30]. Based on the scale proposed by Van Vuuren and Holl, the essential oil of leaves from limoncillo presented a strong activity against the fungus *Aspergillus niger* (ATTC 10231) and Gram-positive bacterium *Enterococcus faecium* (ATCC 27270) with a MIC of 250 μg/mL and 500 μg/mL, respectively. Additionally, the essential oil from the leaves presented a moderate activity against Gram-positive bacteria *Enterococcus faecalis* (ATCC 19433) and *Staphylococcus aureus* (ATCC 25923) and fungus *Candida albicans* (ATTC 10231) with a MIC of 1000 μg/mL. The essential oil of the fruits reported a moderate activity against fungi *Candida albicans* (ATTC 10231) and *Aspergillus niger* (ATTC 10231) with a MIC of 1000 μg/mL. The strong antimicrobial activity against *Aspergillus niger* and *Enterococcus faecium* of the essential oil from leaves may be due to its high concentration in pinene <α-> which have been reported as compounds with antibacterial capacity [31,32]. Although, the moderate essential oil activity of the fruits may be due to the presence of limonene [33]. However, it should be considered that the biological activity in essential oils is a phenomenon that is difficult to predict, although this activity is based on the major and minor compounds of the essential oils, it must be considered that there are synergisms and antagonisms that can cause loss or increase in activities [34]. Further, currently some studies have shown that different enantiomers of a compound have different biological activities. Lis-Balcnin et al. reported that bacteria were more affected by the (−)-α-pinene compared with the (+)-α-pinene and that Listeria monocytogenes and filamentous fungi were more affected by (+)-α-pinene isomer. Silva et al. established that the (+)-α-pinene isomer exerted a microbicidal effect against the fungi and bacteria tested, while with (+)-α-pinene no activity was observed [35]. Omran et al. determined that (−)-limonene had better antifungal activity than (+)-limonene against *Aspergillus niger*, *Candida albicans, Aspergillus* sp., and *Penicillium* sp. [36]. The minimum inhibitory concentration values against four Gram-negative and three Gram-positive bacteria were in the ranges of 2 to 27 mg/mL for (−)-limonene and 3 to 27 mg/mL for (+)-limonene [37].

The essential oil of the leaves and of the fruits showed a moderate antioxidant activity by the ABTS method, by the DPPH method the essential oil of the leaves showed a weak activity and with the essential oil of the fruits it was impossible to obtain the SC_50_ at the maximum concentration. tested (4000 µg/mL). The weak antioxidant activity in the DPPH method may be explained by the fact that terpene compounds are incapable of donating a hydrogen atom [38], so that, ABTS method is more appropriate to determine antioxidant activity of lipophilic substances, such as essential oils [39].

The acetylcholinesterase inhibitory activity from leaves and fruit of *Siparuna muricata* has not been reported previously. *Siparuna muricata* leaves and fruits essential oil showed an IC_50_ value of 52.98 ± 1.04 µg/mL and 98.84 ± 1.04 µg/mL), respectively, this activity could be considered as compelling compared to activities of several piper species reported in bibliography (IC_50_ of 12.4 to 1.5 mg/mL) [40,41]. The α-pinene, a main compound in the oils in our study, has been found to be the major compound in essential oils with high anticholinesterase activity [42]. Cholinesterase is a term referring to one of two enzymes, named, acetylcholinesterase (AChE) found mainly in blood and nerve synapses, and pseudocholinesterase found mainly in the liver. The difference between the two types of cholinesterase is in their respective preferences for substrates, acetylcholinesterase hydrolyzes acetylcholine faster, and pseudocholinesterase hydrolyzes butyrylcholine faster. Both compounds catalyze the hydrolysis of excess neurotransmitter acetylcholine (ACh) in the synaptic gap into choline and acetic acid, a reaction necessary to allow the cholinergic neuron to return to its resting state after activation, thus avoiding excessive activation caused by acetylcholine, which would produce an overstimulation of the effector and, as a consequence, damage to the neuron or muscle [43]. A cholinesterase inhibitor is known as an anticholinesterase compound. Anticholinesterase are also used for treating myasthenia gravis, glaucoma, and Alzheimer’s disease [44]. Because of anticholinesterase essential role, the chemicals that interfere with cholinesterase action are potent neurotoxins and considering the drawbacks of synthetic inhibitors of acetylcholinesterase including gastrointestinal disturbances, moderate effectiveness, high cost, and short half-life [45], compounds isolated from natural products (plants) have been increasingly explored for better effects [46,47]. Essential oils exhibit pharmacological properties traceable to the presence of several structurally diverse bioactive chemical constituents and are increasingly being exploited for their anticholinesterase properties [48].

## 4. Materials and Methods

### 4.1. Materials

2,2′-azinobis-3-ethylbenzothiazoline-6-sulfonic acid (ABTS), 2,2-diphenyl-1-picrylhydryl (DPPH), 5,5′-dithiobis (2-nitrobenzoic acid) (DTNB), acetylcholinesterase (AChE), acetylthiocholine (AcSCh), butylated hydroxytoluene (BHT), dichloromethane (DMC), dimethyl sulfoxide (DMSO), donepezil, methanol (MeOH), magnesium chloride hexahydrate, phosphate buffered saline (PBS), tris hydrochloride (Tris-HCl) and sodium sulfate anhydrous were purchased from Sigma-Aldrich (San Luis, MO, USA). Mueller Himton broth, Mueller Hinton II broth and fluid thioglycollate medium were purchased from DIPCO (Quito, Ecuador). The standard aliphatic hydrocarbons were purchased from ChemService (West Chester, PA, USA). Helium was purchased from INDURA (Quito, Ecuador). All chemicals were of analytical grade and used without further purification.

### 4.2. Plant Material

The leaves and fruits of *Siparuna muricata* were collected in Villonaco hill (4°00’11” S and 79°15’31” W), canton and province of Loja at 2725 m a.s.l. The plant was collected under permission number MAE-DNB-CM-2016-0048 of the Ministerio del Ambiente, Agua y Transición Ecológica of Ecuador. Airtight plastic containers were used for storage and transfer of the plant material until its use. The collection and transfer pressure were approximately 80 KPa (room pressure) and the temperature was 16–18 °C (room temperature). The identification of the plant material was carried out by the curator of the herbarium of Universidad Técnica Particular de Loja (HUTPL). A voucher specimen was deposited at the HUTPL under the code HUTPL14228.

### 4.3. Essential Oil Isolation

The isolation of essential oil from the leaves and fruits of *Siparuna muricata* was carried out according to the procedure described by Valarezo et al. [49] using a Clevenger-type apparatus. The samples were subjected to hydrodistillation for 3 h. After the essential oil obtained were decanted, dried using anhydrous sodium sulfate and stored at 4 °C in amber sealed vials until being used in analysis.

### 4.4. Identification and Quantification of Essential Oil Compounds

The compounds identification was done quantitatively and qualitatively according to the procedures described by Valarezo et al. [50]. Quantitative analyses were performed by gas chromatography equipped with a flame ionization detector (GC-FID) using an Agilent gas chromatograph (GC) (model 6890N series, Agilent Technologies, Santa Clara, CA, USA) and a nonpolar Agilent J&W DB-5ms Ultra Inert GC column (30 m, 0.25 mm, 0.25 µm) with stationary phase 5%-phenyl-methylpolyxilosane. The relative amounts of individual components were calculated based on the chromatographic peak area of the flame ionization detector response, without using a correction factor. Qualitative analyzes were performed with a gas chromatography coupled to mass spectrometer (GC-MS) using, the same chromatograph described for GC-FID, but in this procedure, it was coupled to a mass spectrometer (quadrupole) detector (MS) (model Agilent series 5973 inert, Agilent Technologies, Santa Clara, CA, USA). The identification of the oil components was based on a comparison of mass spectrum and relative retention indices (RI) of the compounds with those of published literature [51,52]. RI was determined using a standard of aliphatic hydrocarbons (C_9_ to C_25_). The RI was obtained with the equation 1 [53]

(1) $$\mathrm{RI}=100n+\frac{\left(\mathrm{RTx}-\mathrm{RTn}\right)}{\left(\mathrm{RTN}-\mathrm{RTn}\right)}$$

where n and N are the carbon number of the hydrocarbon that elutes before and after the compound of interest, respectively, RT is the retention time and RTx is the retention time of the compound of interest.

### 4.5. Enantioselective Analysis

Enantioselective analyses were performed, according to the procedures described by Cartuche et al. [54], using gas chromatography (Trace 1310, Thermo Fisher Scientific, Waltham, MA, USA) coupled to a mass spectrometer (quadrupole) (ISQ 7000, Thermo Fisher Scientific, Waltham, MA, USA) and an enantioselective GC column (MEGA-DEX DMT-Beta, Mega, Legnano, MI, Italy) with stationary phase 2,3-diethyl-6-tert-butyldimethylsilyl-β-cyclodextrin. The elution order of the enantiomers of the compounds was determined based on the technical data of the column. The enantiomeric excess was calculated as the percentage of the major enantiomer minus the percentage of the minor enantiomer.

### 4.6. Antimicrobial Activity

Antibacterial and antifungal activity of essential oil were determined by means of the broth microdilution method according to the procedures described by Cartuche et al. [54]. For the antimicrobial activity, the Gram-positive bacteria *Enterococcus faecalis* (ATCC 19433), *Enterococcus faecium* (ATCC 27270) and *Staphylococcus aureus* (ATCC 25923) and the Gram-negative bacteria *Escherichia coli* O157:H7 (ATCC 43888) and *Pseudomonas aeruginosa* (ATCC 10145) were used. The fungi *Candida albicans* (ATCC 10231) and *Aspergillus niger* (ATCC 10231) were used to determine the antifungal activity. The Minimal inhibitory concentration (MIC) was used to report the values of the activity. DMSO was used as a negative control and ampicillin for Gram-positive bacteria, ciprofloxacin for Gram-negative bacteria and amphotericin B for fungi were used as a positive control.

### 4.7. Evaluation of Antioxidant Capacity

The antioxidant capacity of the essential oil of the leaves and fruits of *Siparuna muricata* was evaluated with the ABTS and DPPH methods according to the procedure described by Cartuche et al. [54]. In the ABTS method, the reagent 2,2’-azinobis (3-ethylbenzothiazoline-6-sulfonic acid) (ABTS) was used to produce the 2,2’-azinobis(3-ethylbenzothiazoline-6-sulfonic acid) radical cation (ABTS^•+^), which was used to evaluate the free radical scavenging of the two essential oils. In the DPPH method the reagent 2,2-diphenyl-1-picrylhydrazyl (DPPH) was used to produce the 2,2-diphenyl-1-picrylhydrazyl radical (DPPH^•^), which was used to measure the free radical scavenging activity of essential oils. In both methods, a UV spectrophotometer (Genesys 10S UV-Vis Spectrophotometer, Thermo Fisher Scientific, Waltham, MA, USA) was used to measure the absorbance of the samples at a wavelength of 734 nm and 515 nm for the ABTS and DPPH method, respectively. The antioxidant activity of the essential oils was expressed as SC_50_, which is the concentration value necessary for the essential oil to have half radical scavenging capacity. Trolox was used as a positive control and methanol as a negative control

### 4.8. Anticholinesterase Activity

The acetylcholinesterase inhibitory effect of the essential oil from the leaves and fruits of *Siparuna muricata* was measured according to what was described by Valarezo et al. [49] using a microplate spectrophotometer (EPOCH 2, BioTek, Winooski, VT, USA) at a wavelength of 405 nm. The IC_50_ (concentration of essential oil required for 50% inhibition) value was measured from the corresponding rate of the reaction curve. Methanol was used as a negative control and donepezil hydrochloride as a positive control.

### 4.9. Statistical Analysis

The analyses of essential oil isolation, evaluation of antioxidant capacity and anticholinesterase activity were performed in triplicate. The procedures of identification of essential oil compounds, enantioselective analysis and antimicrobial activity were performed nine times. Data were collected in Microsoft Excel, measures of central tendency and standard deviation were calculated using Minitab 17 (Version 17.1.0., Minitab LLC., State College, PA, USA). All results are expressed as mean values.

## 5. Conclusions

The chemical composition, enantiomeric distribution, and biological activity of essential oil from fruits of *Siparuna muricata*, as well as the biological activity of essential oil from leaves of this species were determined for the first time; in the leaf essential oil were identified 51 compounds and 41 in the fruit oil. The main compounds was α-pinene in the essential oil of the leaves and limonene in the fruit oil. In both essential oils five pairs of enantiomers were identified. Additionally, it was determined that the essential oil from the leaves of *Siparuna muricata* presented a strong activity against *Aspergillus niger* and *Enterococcus faecium* and that the essential oil from fruits and leaves presented a moderate antioxidant activity and very strong anticholinesterase activity. This research contributes to the knowledge of Ecuadorian biodiversity and presents new opportunities for the exploitation of native and endemic aromatic species. Biological activities of the *Siparuna muricata* leaves and fruits make this species novel for the food, cosmetic, and pharmaceutical industries. Based on the promising results of the in vitro activity, it is proposed that future studies perform the in vivo activity, such as the anti-inflammatory activity in mice.

## Figures and Tables

**Table 1:** Chemical composition of essential oil from the leaves and fruits of *Siparuna muricata*.

N°	Compound ^a^	RIC	RIR	Leaves			Fruits			CF
				%		SD	%		SD	
1	Pinene <α->	932	932	23.22	±	1.03	10.90	±	1.79	C_10_H_16_
2	Camphene	949	946	5.17	±	0.14	2.29	±	0.22	C_10_H_16_
3	Thuja-2,4(10)-diene	953	953	0.41	±	0.04	0.36	±	0.05	C_10_H_16_
4	Sabinene	975	969		-		3.63	±	0.15	C_10_H_16_
5	Pinene <β->	978	974	9.47	±	1.33		-		C_10_H_16_
6	6-Methyl-5-hepten-2-one	989	981		-		0.94	±	0.04	C_8_H_14_O
7	Menthene <3-ρ->	989	984	0.28	±	0.01		-		C_10_H_18_
8	Myrcene	990	988	2.17	±	0.12	0.90	±	0.08	C_10_H_16_
9	Mentha-1(7),8-diene <ρ->	1004	1003		-		0.18	±	0.08	C_10_H_16_
10	Hexyl acetate	1008	1007		-		1.86	±	0.09	C_8_H_16_O_2_
11	Cymene <ο->	1027	1022	0.33	±	0.12	0.39	±	0.03	C_10_H_14_
12	Limonene	1030	1024	8.71	±	0.06	24.92	±	1.20	C_10_H_16_
13	ß-Phellandrene	1032	1025	1.1	±	0.05	0.20	±	0.03	C_10_H_16_
14	BicOcimene <(Z)-β->	1034	1037		-		2.08	±	0.05	C_10_H_16_
15	Ocimene <(E)-β->	1037	1044	0.30	±	0.01		-		C_10_H_16_
16	Guaiacol <ο->	1093	1087	0.30	±	0.01		-		C_7_H_8_O_2_
17	Perillene	1102	1102	0.53	±	0.08		-		C_10_H_14_O
18	Linalool	1105	1095	0.34	±	0.22	4.08	±	0.19	C_10_H_18_O
19	Nonanal <n->	1109	1100		-		0.68	±	0.16	C_9_H_18_O
20	Mentha-2,8-dien-1-ol <trans-p->	1125	1119		-		0.53	±	0.24	C_10_H_16_O
21	Campholenal <α->	1133	1122	0.86	±	0.11	0.51	±	0.24	C_10_H_16_O
22	Pinocarveol <trans->	1143	1135		-		0.61	±	0.22	C_10_H_16_O
23	Verbenol <cis->	1145	1137		-		0.70	±	0.1	C_10_H_16_O
24	Sabinol <trans->	1146	1137	1.43	±	0.08	0.55	±	0.28	C_10_H_16_O
25	Chrysanthenol <cis->	1152	1160	0.32	±	0.08		-		C_10_H_16_O
26	Isopulegol <iso->	1155	1155		-		1.66	±	0.6	C_10_H_18_O
27	Rosefuran epoxide	1173	1173		-		0.47	±	0.17	C_10_H_14_O_2_
28	Mentha-1,5-dien-8-ol <p->	1176	1166		-		1.84	±	0.05	C_10_H_16_O
29	Acetophenone <ρ-methyl->	1182	1179	0.80	±	0.01		-		C_9_H_10_O
30	Dihydro carveol	1199	1192		-		2.82	±	0.28	C_10_H_18_O
31	Myrtenal	1204	1195	0.65	±	0.02		-		C_10_H_14_O
32	Safranal	1205	1197	0.47	±	0.01		-		C_10_H_14_O
33	Decanal <n->	1210	1201		-		4.35	±	0.62	C_10_H_20_O
34	Verbenone	1214	1205		-		0.80	±	0.45	C_10_H_14_O
35	Carveol <trans->	1225	1215		-		0.93	±	0.53	C_10_H_16_O
36	Ocimenone <(Z)->	1218	1226	0.41	±	0.01		-		C_10_H_14_O
37	Carveol cis->	1226	1226		-		0.53	±	0.33	C_10_H_16_O
38	Mentha-1(7),8-dien-2-ol <cis-p->	1229	1227	0.27	±	0.02		-		C_10_H_16_O
39	Neral	1245	1235		-		3.19	±	0.66	C_10_H_16_O
40	(−)-Carvone	1252	1249		-		1.10	±	0.06	C_10_H_14_O
41	Methyl citronellate	1261	1261		-		0.86	±	0.49	C_11_H_20_O_2_
42	Pregeijerene B	1278	1274		-		3.68	±	0.22	C_12_H_18_
43	Bornyl acetate	1288	1283	3.81	±	0.42	2.32	±	1.37	C_12_H_20_O_2_
44	Myrtenyl acetate	1329	1324	0.62	±	0.05		-		C_12_H_18_O_2_
45	Elemene <δ->	1334	1335	0.27	±	0.03		-		C_15_H_24_
46	Longicyclene	1369	1371		-		0.11	±	0.08	C_15_H_24_
47	Copaene	1374	1374	0.53	±	0.17	0.62	±	0.69	C_15_H_24_
48	Elemene <β->	1389	1389	0.37	±	0.14		-		C_15_H_24_
49	Longipinene <β->	1409	1400	0.49	±	0.01		-		C_15_H_24_
50	Caryophyllene <(E)->	1418	1417	0.77	±	0.08		-		C_15_H_24_
51	Himachalene <α->	1450	1449	0.39	±	0.06		-		C_15_H_24_
52	Sesquisabinene	1455	1457	0.39	±	0.16		-		C_15_H_24_
53	Aromadendrene <allo->	1461	1458	1.05	±	0.07	0.48	±	0.37	C_15_H_24_
54	Chamigrene <β->	1479	1476	0.42	±	0.11		-		C_15_H_24_
55	Patchoulene <γ->	1498	1502	0.54	±	0.26	0.17	±	0.06	C_15_H_24_
56	Himachalene <β->	1500	1500	0.51	±	0.42		-		C_15_H_24_
57	Bisabolene <β->	1511	1505	1.32	±	0.01		-		C_15_H_24_
58	Amorphene <δ->	1519	1511	0.52	±	0.09		-		C_15_H_24_
59	Zonarene	1522	1528	0.44	±	0.27		-		C_15_H_24_
60	Cubebol <10-epi->	1529	1533	0.75	±	0.05		-		C_15_H_24_O
61	Germacrene B	1562	1559	0.75	±	0.2		-		C_15_H_24_
62	Cedrene epoxide <α->	1581	1574	1.07	±	0.81		-		C_15_H_24_O
63	Spathulenol	1583	1577		-		0.49	±	0.51	C_15_H_24_O
64	Davanone B	1573	1564	0.68	±	0.01		-		C_15_H_24_O_2_
65	Caryophyllene oxide	1587	1582	3.01	±	1.06		-		C_15_H_24_O
66	Globulol	1595	1590	1.15	±	0.17		-		C_15_H_26_O
67	Viridiflorol	1599	1592	1.83	±	2.04		-		C_15_H_26_O
68	Ledol	1603	1602	0.67	±	0.28		-		C_15_H_24_O
69	Humulene epoxide II	1617	1608	0.72	±	0.58		-		C_15_H_24_O
70	Eremoligenol	1634	1629		-		0.55	±	0.54	C_15_H_26_O
71	Acorenol <β->	1638	1636	12.71	±	2.91		-		C_15_H_26_O
72	Butyl phthalide <3->	1642	1647	2.94	±	0.5		-		C_12_H_14_O_2_
73	Himachalol	1660	1652		-		0.62	±	0.33	C_15_H_26_O
74	Helifolenol A	1665	1674	0.63	±	0.12		-		C_15_H_24_
75	Allohimachalol	1668	1661	0.19	±	0.01		-		C_15_H_26_O
76	Khusinol	1680	1679	0.19	±	0.01	2.30	±	1.58	C_15_H_24_O
77	Eicosane <n->	1995	2000		-		3.52	±	1.02	C_20_H_42_
Monoterpene hydrocarbons				51.16			45.84			
Oxygenated monoterpenes				8.75			25.07			
Sesquiterpenes hydrocarbons				8.76			1.38			
Oxygenated sesquiterpenes				22.97			3.96			
Diterpenes hydrocarbons				-			3.52			
Others				5.52			10.34			
Total identified				97.16			90.13			

^a^ Compounds ordered according to the elution order; RIC: Calculated Retention Indices; RIR: References Retention Indices; SD: Standard Deviation; CF: Chemical Formula; -: undetermined.

**Table 2:** Chiral compounds present in the essential oil from the leaves of fruits of *Siparuna muricata*.

Enantiomers		Leaves		Fruits	
	RI	ED (%)	e.e. (%)	ED (%)	e.e. (%)
(1R,5R)-(+)-α-Pinene	935	76.99	53.98	-	-
(1S,5S)-(−)-α-Pinene	943	23.01		100	100
(1S,4R)-(−)-Camphene	960	100	100	87.31	74.62
(1R,4S)-(+)-Camphene	964	-	-	12.96	
(1R,5R)-(+)-β-Pinene	993	72.18	44.36	-	-
(1S,5S)-(−)-β-Pinene	999	27.82		-	-
(4S)-(−)-Limonene	1055	34.42	31.16	21.81	56.38
(4R)-(+)-Limonene	1061	65.58		78.19	
(R)-(−)-Linalool	1202	-	-	33.33	33.34
(S)-(+)-Linalool	1209	-	-	66.67	

RI: retention index; ED: enantiomeric distribution; e.e.: enantiomeric excess.

**Table 3:** Antimicrobial activity of essential oil from the leaves and fruits of *Siparuna muricata*.

Microorganism	Leaves	Fruits	Positive Control ^a^
	MIC (µg/mL)		
Gram-positive cocci			
*Enterococcus faecalis* (ATCC 19433)	1000	4000	0.78
*Enterococcus faecium* (ATCC 27270)	500	4000	0.39
*Staphylococcus aureus* (ATCC 25923)	1000	2000	0.39
Gram-negative bacilli			
*Escherichia coli* O157:H7 (ATCC 43888)	>4000	4000	1.56
*Pseudomonas aeruginosa* (ATCC 10145)	>4000	>4000	0.39
Yeasts and sporulated fungi			
*Candida albicans* (ATTC 10231)	1000	1000	0.098
*Aspergillus niger* (ATTC 10231)	250	1000	0.098

^a^ Ampicillin for *Enterococcus faecalis*, *Enterococcus faecium* and *Staphylococcus aureus*; Ciprofloxacin for *Escherichia coli* and *Pseudomonas aeruginosa*; amphotericin B for *Candida albicans* and *Aspergillus niger*.

**Table 4:** Antioxidant activity of essential oil from the leaves and fruits of *Siparuna muricata*.

Sample	ABTS	DPPH
	SC_50_ (µg/mL) ± SD	
*Siparuna muricata* leaves essential oil	963.3 ± 1.6	4000 ± 5.1
*Siparuna muricata* fruit essential oil	775.3 ± 1.3	-
Trolox	23.3 ± 1.1	30.0 ± 1.0

## Data Availability

Not applicable.
